# Trends towards Effective Analysis of Fluorinated Compounds Using Inductively Coupled Plasma Mass Spectrometry (ICP-MS)

**DOI:** 10.1155/2021/8837315

**Published:** 2021-02-02

**Authors:** Ruth Lorivi Moirana, Thomas Kivevele, Josephine Mkunda, Kelvin Mtei, Revocatus Machunda

**Affiliations:** The Nelson Mandela African Institution of Science and Technology (NM-AIST), School of Material Energy Water and Environmental Sciences, P.O. Box 447, Arusha, Tanzania

## Abstract

Increased demand for monitoring and identification of novel and unknown fluorinated compounds (FCs) has demonstrated the need of sensitive fluorine-specific detectors for unknown FCs in both biological and environmental matrices. Inductively coupled plasma mass spectrometry (ICP-MS) is a promising technique for analysis of FCs and has been rated as the most powerful tool in analytical chemistry. However, direct determination of fluorine using this technique is challenged by high ionization potential of fluorine together with spectral and nonspectral interferences which affect the quality of results. To enhance the quality of results, several studies have reported modifications of a conventional ICP-MS analysis procedure on sample preparation, introduction, analysis, and instrument optimization. Therefore, the focus of this study is to discuss different ICP-MS optimizations and future trends towards the effective analysis of FCs using ICP-MS.

## 1. Introduction

Human activities have led to the establishment of a wide variety of fluorinated precursors. Interaction of these precursors with the environment creates novel fluorinated compounds (FCs). Yeung et al. (2016) reported that novel fluorinated chemical structures make up to 60–90% of total FCs in biological and environmental samples [1, 2]. Yamashita et al. (2004) further revealed that organisms found at the bottom of the food chain have at least higher concentrations of organofluorines which can neither be detected by high-performance liquid chromatography-electrospray ionization-mass spectrometry (HPLC-ESI-MS) nor ionizable by atmospheric pressure chemical ionization (APCI) [3]. The ESI-MS technique exhibits a challenge with a selective nontargeted analytical system as data cannot be extracted magnificently by the mathematical algorithm to identify a given number of F atoms.

Determination and quantification of biologically and environmentally essential ions have significantly caught the attention of researchers in the field of analytical chemistry. This has made fluorine (F), which has certain unique properties such as the smallest radius, hard Lewis basic nature, and high charge density, one of the most extensively studied anion [4]. Additionally, F determination is crucial to contemporary environmental analysis due to its wide natural occurrence and anthropogenic synthesis of its precursors [5].

Chromatographic [6], spectrometric [7], electroanalytical [8], titrimetric [9], and potentiometric [10] techniques have successfully been used for the determination and quantification of FCs in matrices [11]. Other methods such as radiochemical, enzymic, polarographic, and gravimetric have also been utilized, although not prevalently [12]. The chromatographic method [6] comprises ion chromatography (IC), liquid chromatography (LC) (also referred to as high-performance liquid chromatography (HPLC)), and gas chromatography (GC). Whereas spectrometric techniques involve inductively coupled plasma mass-spectrometry (ICP-MS), inductively coupled plasma-optical emission spectrometry (ICP-OES) [11], and X-ray fluorescence spectroscopy (XRF), the electroanalytical method utilizes electrospray ionization (ESI) [13].

Recently, ICP-MS has been utilized successfully for nontargeted fluorine determination and quantification in biological and pharmaceutical samples with limited studies on environmental samples [14]. Determination of FCs with ICP-MS techniques is hampered by the high ionization energy of F together with spectral and nonspectral interferences. These challenges have been overcome for some time now through the modification of conventional analysis procedures of sample preparation, introduction, analysis, and instrument optimization [15]. Therefore, this study discusses trends towards achieving effective nontargeted analysis of FCs using ICP-MS.

## 2. The Occurrence of Fluorinated Compounds (FCs)

Fluorine was first found in human sera in 1966 and was assumed to originate from drinking water [16]. Two years later, it was discovered that human blood contains organic and inorganic FCs which were presumed to originate from environmental aspects of water, air, and soil [17]. The F and its compounds occur naturally in the environment through volcanic activities and weathering of bed-rock material. These activities release F into the soil, water, and air in different forms depending on the nature of the parent-rock material [18]. Anthropogenic activities such as industrialization and fertilizer applications also release FCs of different forms such as polymers and other precursors that react with the naturally occurring F in the environment to form novel FCs [19]. These novel FCs could be toxic to plants and animals including humans. Nonetheless, due to the limitations of analytical abilities, it was almost impossible to identify specific organic F [20]. With recent advances in analytical methods, specific forms of organic F particularly perfluorinated compounds (PFCs), perfluorooctanoate (PFOA), and perfluorooctanesulfonate (PFOS) have been identified in blood and environmental samples [21]. The number of novel FCs identified has augmented to more than 15 by 2005 which is startling [22]. Since then, researchers have been working on finding appropriate analytical methods for the identification and quantification of FCs, as it has been put out that PFOS and PFOA constitute only a small amount of FCs existing in the environment [19, 21, 23–26].

## 3. Targeted and Nontargeted Analysis of FCs

Given a massive number of FCs in the environmental and biological matrices, its analysis presents a significant analytical challenge [27]. A limited number of FCs is quantified using liquid chromatography-tandem mass spectrometry (LC-MS-MS) [28], ionic or gas chromatography-mass spectrometry (GC-MS) [29], and high-resolution mass spectrometry (HR-MS) [30]. These techniques, however, cannot capture all FCs due to a lack of authentic standards. The question of how many FCs are missed out during analysis therefore remains a puzzle.

Analytical methods developed for quantification of total F irrespective of the chemical form or molecular weight are used for rapid screening. These methods include combustion ion chromatography (CIC) [31], nondestructive methods (particle-induced gamma-ray emission spectroscopy (PIGES) [32], fluorine-19 nuclear magnetic resonance spectroscopy (19F NMR) [33]), and total oxidizing precursors (total oxidizable precursor (TOP) assay) [34]. Each of the above-mentioned methods captures a specific fraction of FCs in the samples. TOP assay technique guarantees unidentified organofluorines to be associated with PFAs, yet it is incapable of screening emerging FCs that cannot oxidize into accustomed PFAs. Therefore, this method necessitates the addition of authentic standards of PFAs to broaden its analysis capability [27].

On the other hand, the HR-MS data acquisition step is referred to as nontargeted because it is possible to decide on how to use the information provided (i.e., targeted, suspected, or nontargeted). Targeted involves matching with reference standards already available. Suspect screening does not necessitate the standard reference; instead, the precise mass and isotopic pattern from the molecular formula is used to determine the existing molecule. These two are referred to as targeted analysis because of the analysis of mass spectrometer (MS) data that comes after compound information. With nontargeted analysis, MS data come before compound information and therefore intend to disclose the composition of that particular sample. The nontargeted analysis that intends to establish the known and unknown molecular species of a particular element in a sample is referred to as selective nontargeted analysis [35].

Nontargeted analysis of nonmetals such as chlorine (Cl), bromine (Br), and iodine (I) have so far not been exhausted. This is because of challenges such as low sensitivity in the plasma and interferences that lead to erroneous results [25]. Also, Cl and F have high ionization potentials and are therefore not effectively ionized in argon- (Ar-) based plasma; whereas Br is interfered with argides in ICP-MS. Chlorinated compounds have been determined in ESI-MS technique, although its quantification necessitates specific standards [36]. ICP-MS analysis of chlorinated compounds is limited by its high ionization energy and polyatomic interferences. Nevertheless, the use of tandem ICP-MS (ICP-MS-MS) with hydrogen gas (H_2_) as a reaction gas facilitates its analysis at limits of detection in parts-per-million [37]. On the other hand, analysis of Br has been reported to be successful where high-performance liquid chromatography-tandem ICP-MS (HPLC-ICP-MS-MS) is utilized [38].

Initially, more than 4000 organofluorines compounds were identified. Those that were found not biodegradable were banned (≈15 PFCs), and isotopically labeled standards were prepared for their nontargeted analysis using the HPLC-ESI-MS detection technique [19, 39, 40]. Later, researchers speculated the existence of novel PFCs precursors that could not be determined in ESI-MS. This is because authentic standards for the novel precursors were not available, and mathematical algorithm to determine molecular forms of F in an enormous amount of data generated during nontargeted analysis in ESI-MS was lacking [41]. The ESI-MS technique is cost-effective and easy to use but requires stable pH and ionic strength, and its sensitivity is also insufficient for low fluoride (F^+^) concentrations [42]. On the other hand, CIC is challenged with weak F^+^ binding affinity to exchanging ions during the separation process. Weak binding affinity leads to early elution of F^+^ species from the column producing erroneous results [4].

Spectroscopic methods such as atomic absorption spectrometry (AAS), laser-induced breakdown spectroscopy (LIBS), ICP-MS, and ICP-OES offer an indiscriminate analysis of FCs [2, 43]. Due to the nature of F, the strongest atomic absorbance is at the vacuum-UV range beneath 100 nm [44]. This is beyond the working range of ICP-OES, LIBS, and AAS and therefore results in poor sensitivity. Determination of F in plasma-based techniques was therefore rated as almost impossible [43]. The use of the ICP-MS technique for the determination of FCs has been faced with an insufficient amount of F^+^ synthesized due to the high ionization energy potential of F together with spectral and nonspectral interferences. Analytical chemists have employed several techniques during utilization of ICP-MS to facilitate lower limits of detections, high F^+^ concentrations in the plasma, and minimizing interferences while accomplishing selective nontargeted analysis [44] techniques that are discussed in this study.

## 4. Inductively Coupled Plasma Mass Spectrometry (ICP-MS)

**Figure 1:**
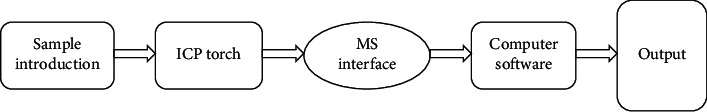
ICP-MS system composition.

ICP-MS is rated as a powerful technique in analytical chemistry (Figure 1). Its advantages over the rest of the ICP techniques are highlighted in Table 1. Determination of F by conventional ICP technique is impaired by its high ionization energy. However, an exception is made to helium- (He-) (ionization energy: 24.6 eV) based plasma. Fluorine (F) is the most electronegative element with ionization energy exceeding that of Ar (the ionization energy of F and Ar is 17, 4228 and 15, 7596 eV, respectively). The high ionization energy of F compared to that of Ar is the cause of the low degree of ionization of F in Ar-based plasma. The combined effect of ionization energy, mass to charge ratio (m/z) (19 for F and 36, 38, and 40 for Ar), and emission lines (nm) (95.187–97.774 for F and 80.286–106.666 for Ar) in low-vacuum UV makes F determination in ICP tools conventionally impracticable [45, 46]. F analysis in ICP-MS is also hampered by spectral and nonspectral interferences which subsequently affect the quality of results as shown in Table 2 [51]. Spectral interferences occur when another species is detected at the same or close to the m/z ratio of the analyte, whereas the nonspectral interference leads to suppression or augmentation of the signal [52]. The latter is also obtained during sample introduction, ion transport from ICP to Ar plasma interface, ion production in plasma, and ion optics to the spectrometer [53, 54].

## 5. FCs Sample Preparation Methods for ICP-MS Analysis

A sample for analysis by ICP-MS is normally diluted using nitric acid or hydrochloric acid to uphold its solubility [55]. Most water samples can easily be analyzed without any preparations. However, solid samples, such as plant material, soil, food, and sludge material, have to be digested in acid and then diluted to the appropriate total dissolved solids (less than 0.5%) and acid concentration [55–57].

Determination of F using conventional quadrupole ICP-MS is challenged by water-derived spectral interferences and tremendously low sensitivity [42]. Therefore, much attention during sample preparation and handling are needed to avoid analysis error. Furthermore, sample preparation before analysis protects the analytical instrument from corrosion, blockage, and contaminants thereby reducing interferences [12, 42, 46]. The disadvantage associated with sample preparation is a change in the composition of the sample. For example, if not handled appropriately during preparation, the sample of interest may change by becoming volatile and may eventually be lost [14].

The majority of FCs contains carbon-based compounds and salts which cause interferences [12]. High concentrations of carbon species in sample solution cause analyte signal augmentation in high and low ICP-MS resolutions due to charge transmission reactions. It is crucial to understand the probable interference before the determination of the procedure for process efficiency [46, 58]. If the interference detection is at the desired limit, then the interferences can be matched with the sample and standard. If the detection limit is not at a critical limit, sample dilution or elimination is achieved to reduce or even remove the interferences completely [59].

Extraction is one of the methods used for the preparation of samples for F analysis. This method involves the use of alkaline solutions such as water-soluble tertiary amine solution (CFA-C), ammonia, tetramethylammonium hydroxide (TMAH), and sodium hydroxide (NaOH) [37, 46]. Other F extraction methods reported involve the use of water [60], organic solvents [25], and acids [61].

Wet digestion is another sample preparation method used to prepare FCs. This method was used to determine F in blood serum, where ether was used as an extract followed by perchloric acid [62]. It is more efficient compared with other methods such as open ashing-fluoride electrode, oxygen-bomb fluorine electrode, and oxygen-bomb gas chromatography in terms of F loss and contamination with extraneous F. The challenge of using this method is the fact that it releases volatile compounds such as HF which causes nonquantitative recoveries [63, 64]. Therefore, the wet digestion method for FCs sample preparation requires novel ways to control the production of HF.

Decomposition methods of combustion and pyrohydrolysis [63, 65] use a concentrated solution [66]. Combustion methods that are used to prepare FCs samples include dry ashing [11, 67, 68], combustion bomb [66, 69–71], Wick-bold apparatus [72], Schoniger flask [73], and microwave-induced combustion [74–76]. These methods are effective; however, they can change sample composition. Methods without matrix decomposition such as dilution, filtration, and solubilization have also been used for the determination of FCs by ICP-MS [77]. These methods are fast and easy, reducing the time spent for sample preparation [47, 49, 50, 77], although they are prone to interferences and contamination of the plasma and nebulization system [25].

Currently, the commonly used FCs sample preparation methods are solid-phase extraction (SPE) and liquid-liquid extractions such as ion-pair extraction (IPE). Water samples are preferably extracted using SPE because it is fast, consumes less organic solvents, and integrates extraction and clean-up [78, 79]. Initially, C_18_ SPE cartridges were used for the preparation of FCs samples; however, its use lately has narrowed due to the presence of trace polyfluorocarbon concentrations in its wrapping materials which tamper with F determination [3]. Weak anion exchange SPE cartridge (WAX) [56] is reported to produce the best extraction for most of FCs; whereas lipophile balance cartridge (HLB) [80] showed efficient extractions of few FCs. Semivolatile and volatile FCs are extracted using an emerging solid-phase microextraction method (SP-ME); however, this method requires derivatization because F tends to augment at the top of the sorbent [13, 81, 82]. IPE employs reagents such as NaOH, methyl tertbutyl ether (MTBE), and tetrabutylammonium hydrogen sulfate (TBA) to extract FCs [83–85]. The FCs dissolve in alkali solution then reacts with TBA which can then be easily extracted by low polar solvent MTBE. IPE has an advantage that it can be used for a variety of samples as compared to SPE which is applied for liquid samples only [86].

Samples in a solid state such as soil, sludge, sediment, and plant are extracted using accelerated solvent extraction (ASE) [28] and ultrasonic extraction (UAE) [87]. The former method provides high efficiency under high temperature and pressure settings; however, it is uneconomical and therefore less preferred [88, 89]. For analysis of plant samples, preparation for F determination mixes several solvents due to the complex matrix composition. A single solvent does not provide efficient extraction. Yoo et al. (2011) used a solvent mixture of dichloromethane-methanol (50 : 50 v/v) and methanol (99 : 1 v/v) and ammonium hydroxide for plant samples extractions; the results were satisfactory [90] and were later supported with Blaine and coworkers who successfully used the same method [91].

## 6. Sample Introduction into ICP-MS

The nebulization process generates water mist during sample introduction which releases ^18^OH^+^ ions. These ^18^OH^+^ ions interfere with both detection and ionization of ^19^F^+^ ion. A low-flow self-aspirating PFA nebulizer and a PFA spray chamber were investigated in hot plasma conditions to overcome the two challenges during sample introduction into the plasma for F determination in high resolution-ICP-MS (HR-ICP-MS). The optimum nebulizer flow rate for a high ionization potential of F was achieved at 0.8 L/min. High gas-flow rate results in chill/cool plasma which affects F ionization. Hot plasma and low flow rates offer ample time for ionization of F. Moreover, fixed nebulizer gas flow rate at a higher radio frequency (RF) power leads to a hotter plasma and therefore favors F determination [47]. Another attempt to overcome the two challenges was reported by Yasuaki Okamoto (2001) who used electrothermal vaporization-ICP-MS (ETV-ICP-MS) for sample introduction. In this technique, the sample insertion port is left open to allow liquid escape when the sample is heated at 200°C for 80 s. When the sample has dried, the port is closed and the temperature is raised to 1100°C for vaporization. The cloud generated containing F^+^ is instantly transported to the ICP by Ar gas. With this technique, the interfering ^18^OH^+^ ions were degraded, and the limit of detection (LOD) of 0.29 *μ*g was attained. The study also suggested increasing the RF power up to 1.4 kW to decrease integrated F^+^ signals [48]. Lower RF power normally used in the nebulization system to eliminate molecular ions was not recommended for use as it leads to unstable plasma discharge and increased baseline as discussed in detail in the review [92].

## 7. Mass Analyzers

The main interferences encountered during quadrupole-ICP-MS (ICP-QMS) application for analysis of FCs are isobaric, doubly charged, and polyatomic [63]. Isobaric interferences occur when signals of different ionic species with the same m/z ratio overlap. To reduce isobaric interferences, isotopes with high natural abundance are preferred. This is because the abundance of the isotope determines the precision of its measurement [46]. The advantage is the fact that most elements have isotopes with unique mass numbers aiding their determination and quantification. Doubly charged interference results when the ions of an element exist with double rather than a common single positive charge [12, 25].

The formation of molecules in the plasma results in polyatomic interference [63]. This is when Ar reacts with elements such as hydrogen (H), nitrogen (N), and oxygen (O) amongst others originating from acids dissolving the samples. Amongst the reported F spectral interferences are ^38^Ar^2+^, ^1^H^18^O^+^, and ^1^H_2_^16^O^1^H^+^ species [43, 77]. Plasma-based instruments have been modified to minimize these interferences. Amongst them are torch change [64], change of interface design, development of dynamic reaction cell technology [50, 93], and hyphenation of ICP-MS with chromatographic methods [49].

The HR-ICP-MS was developed to minimize interferences where interference-free ^19^F^+^ was feasible at a resolution of m/△m ≈ 4000 [43, 47]. Also, a high-resolution double-focusing sector field (HR-ICP-SF-MS) was used by Jakubowski et al. (1998) where high-resolution devices combined with high sensitivity and low background signals offered interference-free analysis [94]. Both of these techniques lessened interferences due to mass overlap, although factors such as operation cost, time, and complexity limit its use for F determination [15, 42, 43].

In response to polyatomic interferences, efforts of producing metal-fluoride {M-F}^+^ ions to determine FCs has been made by separating produced ions from other polyatomic ions using ICP-MS-MS [43]. To separate the produced {M-F}^+^ ions from other polyatomic ions formed by the reaction and collision between ions present in the plasma, an understanding of their formation time is of importance. However, this depends on the energy required to dissociate their bond. Ions with small dissociation energy requirements disintegrate at high temperatures while those with high dissociation energy requirements remain stable at higher temperatures [14, 95]. Therefore, if a molecular ion containing F^+^ with first and second low ionization and high dissociation energy is formed in the plasma, the ICP-MS with a triple quadrupole device (ICP-QQQ) is employed to lessen interferences [4, 59].

Aluminum (Al^3+^) was used to determine FCs in ICP-QQQ by developing {Al-F_3_}^+^ complex. Later, this was separated into AlF^2+^ and Al^3+^ in the ion-exchange column. Although the technique was fruitful, it encountered interferences from Mg^2+^, Zn^2+^, and Fe^2+^. The use of ethylenediaminetetraacetic acid (EDTA) to eliminate Fe^2+^ interference was successful with a LOD of 0.1 ng/mL [42]. Other studies [43, 95] attempted to mix barium (Ba) solution with F solution to generate {Ba-F}^+^ ion in the plasma. This assumption was based on the fact that {Ba-F}^+^ has a low first and second ionization energy of 5.21 and 10 eV, respectively, and high dissociation energy of 6.39 eV. 019 studied a nontargeted analysis of perfluorinated organic compounds (POCs) using ICP-QQQ through mixing Ba solution with an F-containing solution. The researchers monitored the signals of {Ba-F}^+^ at m/z 157. The polyatomic interferences from ^138^Ba-OH^+^ (^138^Ba^18^O^1^H^+^, ^138^Ba^17^O^1^H_2_^+^, and ^138^Ba^16^O^1^H_3_^+^) and ^157^Gd^+^ at the target m/z 157 were prominent; however, they were most pronounced from ^138^Ba-OH^+^. To minimize ^138^Ba-OH^+^ interferences and attain a LOD of 0.05 mg/L, a mass-shift approach was proposed [50].

A mass-shift approach enhances the capability of ICP-QMS to filter stable ions from unstable ones. Oxygen (O_2_) and ammonia (NH_3_) are used as reaction gases in the ICP-QMS [43]. Its use revealed that O_2_ reacts with ^138^Ba-OH^+^ and NH_3_ with {Ba-F}^+^ to form ^138^Ba^19^ F(^14^NH_3_)_3_^+^. The formed new molecules constitute high mass which enhances its separation from the interfering species and hence passes through the detector unaccompanied [43, 49]. To further enhance selectivity, one quadrupole can be implanted upstream of the collision/reaction cell to allow ions of only one m/z to enter the cell. This prevents the generation of new interferences. Guo et al. (2020) considered the economics of using ICP-QQQ as it was far more expensive to be found in the majority of laboratories. These researchers used a single quadrupole-ICP-QMS to determine total F concentration. When online, Ar dilution of the sample before reaching the ICP was used in a conventional ICP-QMS, {Ba-F}^+^ signal dropped 4.6 times, and background signal interferences from ^138^Ba-OH^+^ were lessened by approximately 40-fold [96].

Hyphenation of ICP-MS with HPLC for nontargeted analysis of unknown elemental compounds in matrices has been used for quite some time now. High thermal energy in the plasma enables ICP-MS to handle the nonvolatile mobile phase from HPLC [56]. Determination of FCs by HPLC-ICP-MS necessitates the production of fluoride-polyatomic ions such as {Ba-F}^+^ [49] and {Al-F}^+^ [42] and has the ability to remove emerging interferences through the application of the reaction/collision gas. The first study to analyze FCs using HPLC-ICP-MS was conducted by Jamari et al. (2017). In this study, the formation of fluoride-polyatomic ions of {Ba-F}^+^ was established for its determination in HPLC-ICP-MS/ESI-MS in the presence of O_2_ as a reaction gas and RF power of 1500 W. Following the use of O_2_, interferences from other barium polyatomic were minimized and thus enabled F^+^ determination. The LOD and limit of quantification (LOQ) observed were 0.022 and 0.085 mg/L, respectively, for F and 0.11 and 0.18 mg/L, respectively, for fluoroacetate [50].

Another study employed HPLC-ICP-MS/ESI-MS for identification and quantification of FCs together with their methyl esters as their degradation products. Two separation techniques were investigated: isocratic 70% methanol and acetonitrile/water. F^+^ was determined as a polyatomic ion of {Ba-F}^+^ at 157 *m*/*z*. The organic mode of ICP-MS was used with parameters of the column C_18_ amide and optimized temperature 40°C. The results from HPLC-ICP-MS chromatograms revealed additional peaks of methyl esters initially not detected in ESI-MS. The use of O_2_ as a reaction gas could not eliminate carbon interferences but rather augmented interferences from ^138^Ba-OH^+^ ions. Furthermore, the acetonitrile technique separated FCs well for both ICP-MS and ESI-MS detection, whereas the isocratic technique produced broad peaks in ICP-MS but did not match peaks in ESI-MS. The LOD varied in range 0.49–0.84 mg/L. However, they could be lowered 5-fold by increasing the injection volume from 20 *μ*g to 100 *μ*g [49].

The benefit of using HPLC-ICP-MS for F^+^ determination lies in its ability to reduce interference from metal ions such as praseodymium (Pr), gadolinium (Gd), and additional Ba found in the sample by separation through HPLC. Quantification of F^+^ using HPLC-ICP-MS yielded results similar to HR-ICP-MS and, therefore, suitable for matrices with multiple FCs [50]. Additionally, analysis of ultratrace levels of F^+^ with the HPLC-ICP-MS detector requires the use of “s-lens” other than the commonly used “x-lens” [13]. Furthermore, the presence of Gd in the sample leads to interferences since Gd has the same m/z as that of {Ba-F}^+^; however, the addition of reaction gas such as O_2_ reacts with Gd to form GdO^+^ with m/z 173 which creates a mass shift [50].

An effort to detect F^−^ using ICP-QMS operating in the negative mode when the gas flow rate in the nebulizer was adjusted in the range of 0.6-0.7 L/min, provided the LOD 0.4 mg/L [7, 97]. Towards this mode, better F^−^ detection was observed than in most positive modes, notwithstanding challenges such as lack of collision high background and reaction cells. Therefore, with today's technology of ICP-QQQ, a negative mode of ICP-QMS could be used to lessen background polyatomic interfering ions [13].

## 8. Calibration, Precision, and Accuracy

The matrix effects in ICP-QMS are corrected by the addition of reaction cells which can be complicated and uneconomical. An alternative to this arrangement is the use of calibration strategies which can be executed easily in an economical manner. The calibration strategies reported for the analysis of FCs are external (EC) [43, 77] and internal calibration (IC) [19, 50].

Gou et al. (2017) used multiple-point EC during the determination of total F in food and tea samples using ICP-MS-MS. A series of F standard solutions ranging from 0.1 to 10 *μ*g/mL was used to draw a calibration curve and then obtain a linear correlation coefficient (*R*^2^) = 0.9999. The accuracy of this method was dignified based on calculating the statistical difference between the witnessed values on the proposed method with those certified on purchased food-related standard reference materials (SRMs) [43]. Although EC is vastly preferred due to its simplicity, its use for complex matrix samples leads to inaccurate results. Bader (1980) reported EC to be unable to produce desired results when the calibration standard solution is different from the sample solution. Also, other factors such as pH, ionic strength, physical-chemical properties, and temperature can produce inaccurate results even when total digestion is used for sample preparation [98]. The abovementioned challenges were observed during the analysis of other compounds but were not reported for FCs. Therefore, further studies to investigate the influence of the abovementioned challenges during the use of EC for the analysis of FCs are necessary.

The IC is useful because it enables the determination and quantification of new peaks in chromatograms. This advantage favors its application for nontargeted analysis arrangements. Jamari et al. (2019) used the IC method for nontargeted analysis of PFCs using HPLC-ICPMS/MS-ESI-MS where two standards were spiked into the samples at varying concentrations. The calibration graphs generated revealed the ability of ICP-MS-MS to yield compound unspecific detection (nontargeted); however, this depends on a separation method used (the isocratic elution method is efficient) [50]. Similar results were also obtained from other studies including HR-ICP-MS [47, 49, 99].

While most studies did not give a reason as to the choice of an internal standard (IS) during IC, two main factors are of importance when selecting an IS. First, the analyte and IS should have comparable intensities and therefore signals, and second, both the analyte and IS should have the same physical-chemical properties so that they both undergo the same evolutions during analysis [100]. Some of the IS used for IC in the analysis of FCs include fluoride [47, 49], perfluorohexanoic acid (PFHxA), perfluorooctanoic acid (PFOA) [50], and PFOS and PFOA [99]. However, an insignificant amount of fluorine and shorter carbon chain IS for FCs leads to a smaller response as compared to long carbon chained due to differences in the transport efficiency within the plasma [50].

## 9. Conclusion

A supreme F analytical method should be able to perform a nontargeted determination and quantification with minimal or without sample prepreparation procedures efficiently. Most F analytical methods employed today necessitate extensive sample preparation and yet incapable of nontargeted analysis. The use of ICP-MS has demonstrated the ability to perform nontargeted analysis at low LOD successfully with the advancement in instrumentation and methodology. Although it still entails extensive sample preparation methods to reduce interferences during analysis, yet it is a promising technique. Hyphenation of ICP-MS to HPLC proved to be the most efficient method for the determination and quantification of FCs, particularly for interference minimization. Notwithstanding, little has been done on environmental samples. Also, the current advancements in the determination and quantification of FCs in the positive mode of ICP-MS could be explored in the negative mode of ICP-QMS for analysis of FCs.

## Figures and Tables

**Table 1:** Comparison between OES, AAS, and ICP-MS [11].

	AAS	ICP-OES	ICP-MS
Elemental analysis	Identifies a single element at a time	Capable of identifying the amount of multiple elements in a single sample	Capable of identifying the amount of multiple elements in a single sample
Limit of detection	Good limits of detection for many elements	Better than AAS	Provides the lowest limits of detection
Working range	2 orders of magnitude	Up to 6 orders of magnitude (1 *μ*g/L–1 g/L)	9 orders of magnitude (1 ng/L–1 g/L)
Cost	Low	Medium	High
Isotope analysis	No	No	Capable of analyzing isotopes of the same elements

**Table 2:** Trends towards the selective nontargeted analysis of fluorinated complexes in ICP-MS.

Sample	Sample preparation	Nebulizer	Technique	LOD	Analyte	Interferences	Interference corrections	Remarks	Reference
Tea	Microwave digestion	Micromist	ICP-MS-MS	0.022 *μ*g/L	Ba-F^+^ (m/z 157)	^138^Ba-OH^+>157^Gd^+^	O_2,_ NH_3_/He	The sensitivity of^157^Gd^+^ in ICP-MS is 1000 times greater than that of^138^Ba^19^ F^+^ before the addition of NH_3_ and O_2_ as reaction gases	[43]
Seawater	Acid digestion	Cocentric pneumatic	ICP-MS	0.1 ng/mL	Al-F^2+^ (m/z 27)	Mg^2+^ Zn^2+^ Fe^2+^	Long ion-exchange column and EDTA	Presents lowest LOD ever reported in ICP-MS. Fluoride determination is influenced by the excess of aluminum added to the sample.	[42]
Drug	Wet and acid-digestion	PFA microcentric pneumatic	HR-ICP-MS	5 *μ*g/mL	F^+^ (m/z 19)	^1^H_3_^16^O_1_ ^1^H_2_^17^O_1_ ^1^H^18^O_1_ ^38^Ar^2+^	Medium resolution was used (m/△m ≈ 4000)	It does not necessitate extensive sample preparation	[47]
Water	Extraction	ETV	ETV-ICP-MS	3200 *μ*g/L	F^+^*(m/z 19)*	^18^OH^+^ ^38^Ar^2+^	Elevating the temperature in the vaporizer and the sampling depth to 10 mm	The interferences become prominent when the depth from the sampling orifice is insufficient (<10 mm); however, higher depths reduce ^18^OH^+^ interference and increase 19F^+^ intensity. Can be used for gaseous FCs even if the molecules contain oxygen atoms.	[48]
River water	SPE	Micromist pneumatic	HPLC-ICP-MS-MS	22 *μ*g/L for fluoride. 110 *μ*g/L for fluoroacetate.	Ba-F^+^*(m/z 157)*	^138^Ba^16^O^+138^Ba^16^O^1^H^+157^Gd^+^	O_2_, H_2_, O_2_/H_2_	Suitable for analysis of samples containing multiple FCs and quantification of unknown FCs with respect to their fluorine content. HPLC reduces a significant amount of interferences from metal ions.	[49]
Water	SPE	Micromist	HPLC-ICP-MS-MS-ESI-MS	0.49 mg/L for PFAs	Ba-F^+^ (m/z 157)	^138^Ba^16^O^+138^Ba^16^O^1^H^+^	O_2_	The undetectable FCs in ESI-MS were detected in ICP-MS. Can be used to mine ESI-MS data. O_2_ could not eliminate carbon-based interferences. The use of O_2_ increased interferences from^138^Ba^16^O^1^H^+^	[50]
